# Detection of Human Fall Using Floor Vibration and Multi-Features Semi-Supervised SVM

**DOI:** 10.3390/s19173720

**Published:** 2019-08-28

**Authors:** Chengyin Liu, Zhaoshuo Jiang, Xiangxiang Su, Samuel Benzoni, Alec Maxwell

**Affiliations:** 1 Department of Civil and Environmental Engineering, Harbin Institute of Technology, Shenzhen 518055, China; 2 School of Engineering, San Francisco State University, San Francisco, CA 94132, USA

**Keywords:** falling detection, fall loading model, floor vibration, multi-features semi-supervised support vector machines, benchmark problem

## Abstract

Human falls are the premier cause of fatal and nonfatal injuries among older adults. The health outcome of a fall event is largely dependent on rapid response and rescue of the fallen elder. Being able to provide an accurate and fast fall detection will dramatically improve the health outcomes of the older population and reduce the associated healthcare cost after a fall. To achieve the goal, a multi-features semi-supervised support vector machines (MFSS-SVM) algorithm utilizing measurements from structural floor vibration obtained through accelerometers is proposed in this study to detect falling events with limited labeled samples. In this MFSS-SVM algorithm, the peak value, energy, and correlation coefficient of the accelerometer signal are used as classification features. The performance of the proposed algorithm was validated with laboratory experiments among activities including falling, walking, free jumping, rhythmic jumping, bag dropping, and ball dropping. To further illustrate the performance of the algorithm, a benchmark database was adopted and expanded to test its ability to accurately identify falling, compared with the algorithm used in the benchmark study. Results show that by using the proposed algorithm, the falling events can be identified with high accuracy and confidence, even with small training datasets and test nodes.

## 1. Introduction

The world’s population is growing older. According to the recent report from the Census Bureau, it is estimated that 1.6 billion (17%) of the total 9.4 billion population will be 65 and older in 2050 [1]. Human falls are the premier cause of fatal and nonfatal injuries among older adults, which affect one in three aged 65 and over half of those aged 80+ every year [2,3]. The health outcome of a fall event is largely dependent on rapid response and rescue of the fallen elder.

Based on whether the user’s interference is necessary, current fall detection methods can be classified into two categories: user-dependent and user-independent. The user-dependent methods either use wearable devices (e.g., pendant, watch, clip, bracelet, or ring) to track rapid changes in its orientation or rely on users themselves to report the emergency of fall by interacting (e.g., pressing) the wearable device [4,5,6,7,8,9]. These options suffer from limitations such as getting the person to wear the device or false-positive detection (e.g., a fall is incorrectly identified when no fall occurs) due to user’s abrupt movement. Research on fall detection through user-independent means often uses infrared cameras to monitor users’ behavior and detect fall events with computer vision techniques [10,11]. However, the use of cameras would meet with resistance as residents have a feeling of “being watched” [12]. Progress toward new methods without cameras has been made through the use of accelerometer sensors mounted on the structure to capture floor vibration for human activities detection [13,14,15]. For example, Madarshahian et al. [13] proposed a benchmark problem to promote exploration of floor vibrations to classify human activities. The benchmark problem provided 16,100 records of floor vibrations induced by human activities including bouncing a ball, jumping, and dropping a bag and included an example solution with an algorithm based on the difference of the acceleration autocorrelations. Poston et al. [14] attempted to use measurements of footstep-generated vibrations from accelerometers originally deployed to measure a building’s structural dynamics to locate individuals moving within a building. In this study, a specific time-of-arrival estimation algorithm suited to the type of footstep-to-sensor interaction was developed to work with the data collected through 12 single-axis accelerometers mounted underneath the building floor.

For fall detection, in particular, Alwan et al. [12] utilized a special piezoelectric sensor coupled to the floor surface using a mass and spring arrangement together with battery-powered preprocessing electronics to capture the floor vibration. The authors identified the falling events through passively monitoring the floor vibration patterns. Zigel et al. [15] used floor vibration and sound sensing in sequence with signal processing and a pattern recognition algorithm to detect falling events. In general, the detection algorithms can be categorized into threshold-based and classifier-based. In threshold-based algorithms, thresholds are set for various chosen features, to which the measured values will be compared during the detection phase. Due to large variations and uncertainties in actual usages, such as structural configurations, impact locations, and the distance between the impact and the sensors, the thresholds may need to be modified dramatically for different implementations. This creates challenges for determining a set of thresholds for all scenarios. With the advancement of technologies and computational power, the use of classifiers for various applications has received significant attention in recent years. Classifiers, such as k-nearest neighbors [16,17], support vector machines [18,19], naïve Bayes [20], artificial neural networks [21,22], hidden Markov model [23], and fuzzy logic [24], have been investigated to improve the identification accuracy. One of the challenges these classifier-based methods face is the need for a large training dataset.

In this study, a multi-features semi-supervised support vector machines (MFSS-SVM) algorithm with a radial basis function kernel is proposed to identify falling events through the use of accelerometer measurements from floor vibration. The proposed MFSS-SVM algorithm makes use of the unlabeled datasets and multi-feature classifier integrated from several base classifiers during the training process, resulting in an improved performance with fewer required training datasets and shorter training time. To generate the fall datasets, highly-imitated dummies were utilized to mimic human falling. This paper first provides a brief review of SVM, semi-supervised SVM, and multi-features semi-supervised SVM, followed by the introduction of the proposed algorithm. Next, the performance of the proposed algorithm was investigated with laboratory testing and through an expanded benchmark database.

## 2. Proposed Fall Detection Algorithm

### 2.1. SVM Classifier

To correctly detect a human fall, a pattern recognition needs to be performed to differentiate the fall from other daily activities, such as walking, jumping, and impact from an object falling. After extracting the desired information from the raw data, a classifier is typically designed and developed to categorize the different activities during the pattern recognition process. Among various classifiers, neural networks and SVM are two popular strategies for supervised machine learning and classification. The neural network is widely used, given its ability to learn and model both simple and complex relationships. However, selecting the appropriate features may require extensive expertise, and the time needed to train the model could hinder its usage. The SVM has been shown to be a superior method for binary classification [25,26]. For instance, Davis et al. [27] studied the use of SVMs to classify floor vibration signals to differentiate signals of interest.

Typically, the SVM performs the task of binary classification by mapping input vectors into a hyperplane, a high-dimensional feature space, that is constructed to separate input vectors [28]. If it exists, this hyperplane maximizes the margin between different classes, and thus, the well-known generalization measure called Vapnik–Chervonenkis dimension [29]. Simultaneously, the hyperplane minimizes the empirical error, which is the error committed by the training data. Equation (1) is a mathematical representation that can be used to describe the optimization problem of a general SVM classification.

(1) $$minimize\left\{\frac{1}{2}w\cdot w+C\sum_{i=1}^{l}\xi_{i}\right\},\;\mathrm{subject}\;\mathrm{to}\;y_{i}\left(w\cdot \varphi \left(x_{i}\right)+b\right)\geq 1-\xi_{i,}\;\left(i=1,\;2,\;3,\ldots l\right),$$

where C is the penalty parameter and $\xi_{i}$ is the slack variable, a parameter to handle inseparable data.

Generally, maximizing the classification margin and minimizing the misclassifying rate minimizes Equation (1). The parameter C controls the trade-off between the size of the margin and the penalty of slack variables, a measure of the error. $y_{i}$ (i from 1 to l) ∈ ±1 represents the class labels for different training cases and $x_{i}$ represents the independent variables. φ is the kernel function used to transform data from the input vectors into the feature spaces. Linear kernel, polynomial kernel, and radial basis function (RBF) kernel are the three kinds of functions commonly used in practice. In this paper, the RBF is adopted as the kernel functions of SVM.

### 2.2. Semi-Supervised SVM

To the best of the authors’ knowledge, the first semi-supervised SVM method was proposed by Joachims [30] for text classification purpose, and later improved approaches have been presented in the literature for multiple purposes, such as image/text classification [31,32,33] and vison-based fall detection [34,35,36]. While traditional SVM classifiers utilize a general decision function with a set of labeled training samples, a semi-supervised SVM iteratively takes advantage of the unlabeled datasets and simultaneously considers all the available labeled and unlabeled samples to minimize the classification errors. In the initial iteration, an SVM model is first trained using Equation (1) and pseudo-labels are assigned to the unlabeled datasets. Some of these newly labeled samples are further chosen as semi-labeled samples based on a given criterion. From the theory of SVM, only the samples within the margins (i.e., the samples that have the decision function value y between ±1) have influences on the position of the hyperplane that separates the different classification categories. Among these samples, the ones closest to the margins (i.e., y=±1) have the highest probability of being correctly labeled. Thus, the unlabeled samples that are closest to the margins are usually added to the training dataset, resulting in a hybrid dataset with labeled and semi-labeled samples. The optimization problem in Equation (1) now can be converted to:

(2) $$\begin{array}{c} minimize\left\{\frac{1}{2}\|W{\|}^{2}+C\overset{n}{\underset{l=1}{\sum }}\xi_{l}+C^{*}\overset{d}{\underset{u=1}{\sum }}\xi_{u}^{*}\right\}\\ y_{l}\left(W^{T}\cdot \Phi \left(X\right)+b\right)\geq 1-\xi_{l},\;\xi_{l}\geq 0,l=1,\cdots ,n\\ y_{u}^{*}\left(W^{T}\cdot \Phi \left(X^{*}\right)+b\right)\geq 1-\xi_{u}^{*},\;\xi_{u}^{*}\geq 0,u=1,\cdots ,d \end{array}$$

where $\xi_{l}\;and\;\xi_{u}^{*}$ are the slack variables for the labeled and semi-labeled samples, respectively. C and $C^{*}$ are the penalty parameters for the labeled and semi-labeled samples, respectively.

With the new hybrid training set, the semi-supervised SVM is retrained iteratively until it reaches a stopping criterion (e.g., maximum number of iterations). At each iteration, new semi-labeled samples obtained in the previous iteration are added to the training dataset. The semi-labeled samples whose label has changed from the previous iteration are removed from the new semi-labeled dataset and put back to the unlabeled dataset.

### 2.3. Multi-Feature Semi-Supervised SVM Framework for Human Fall Detection

In this paper, the MFSS-SVM is proposed to specifically tackle the human falling classification problem. In particular, compared with the traditional semi-supervised SVM, the unique characteristic of the MFSS-SVM lies in its multi-feature classifier integrated from several base classifiers. Researches in the previous studies have shown that better classification performance can be achieved for greater difference among the base classifiers. In addition to the common features of the time-domain acceleration signals, such as the peak value, two features, namely energy and sensor correlation coefficient, were selected to identify the vibration characteristics of falling in the records.

(1) Peak value: This feature determines the maximum absolute value of the signals. It can be expressed as
  (3) $$A_{max}=max\left(\left|a\left(t\right)\right|\right),$$
  where a(t) is the time history of the acceleration signal.
(2) Energy: The energy metric attempts to characterize the strength of the recorded signal. It can be calculated by integrating the area under the time history of the acceleration signal.
  (4) E=∫|a(t)|dt.
(3) Sensor correlation coefficient: This metric serves as an indication of how close the correlation between two different acceleration signals and can be described as
  (5) $$\rho_{a_{1}a_{2}}=\frac{\mathrm{Cov}\left(a_{1}\left(t\right),a_{2}\left(t\right)\right)}{\sqrt{D\left(a_{1}\left(t\right)\right)}\sqrt{D\left(a_{2}\left(t\right)\right)}}$$
  where $a_{1}\left(t\right)$ and $a_{2}\left(t\right)$ are the acceleration signals from two accelerometers; Cov() is the correlation deviation of $a_{1}\left(t\right)$ and $a_{2}\left(t\right)$; D() is the standard deviation. It is expected that, by taking consideration of the sensor correlations, environmental random effects are reduced to better represent the event.

Compared to statistical features, such as standard deviation, mean value, and Root Mean Square, these proposed features were identified to have higher divergence in a preliminary study and thus adopted in this study to be extracted as classification features in the data pre-processing.

The tri-training cooperative training strategy [37,38] was applied to the three different base classifiers obtained through the three extracted features defined above [39,40,41], after which an agent-based statistical scheme, the majority-vote model [42], was utilized to integrate them to form the MFSS-SVM classifier. The labeling rate of the MFSS-SVM was also investigated in this study to strike a balance between accuracy and speed, as a high number of labeling rate increases computational burden for real-time MFSS-SVM classification.

**Figure 1:**
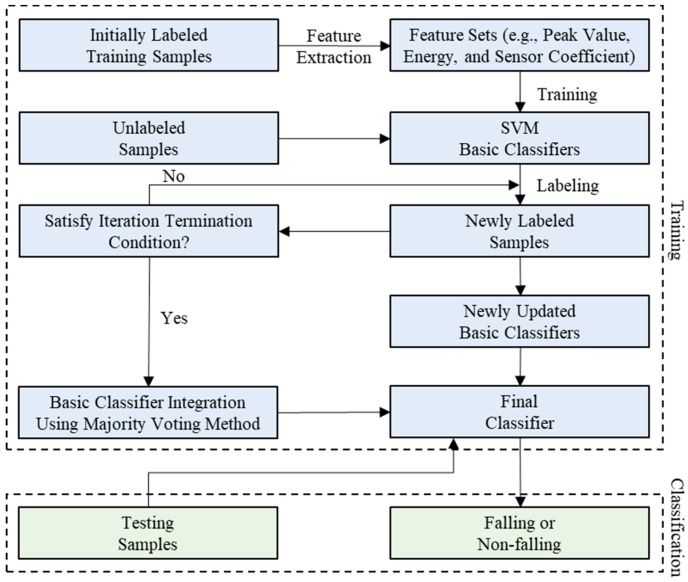
The proposed multi-features semi-supervised support vector machines (MFSS-SVM) framework.

The proposed MFSS-SVM framework, composed of the training and testing phases, is demonstrated in Figure 1. The steps for implementing the framework can be described as

Step 1. Three feature vectors are calculated by extracting peak value, energy, and sensor correlation coefficients from the training samples.

Step 2. The RBF classifier is employed in SVM training on these three extracted features.

Step 3. The tri-training cooperative training strategy is applied to semi-supervised learning.

Step 4. The majority-vote technique is applied to integrate three different basic classifiers resulting from the previous step to form the MFSS-SVM classifier.

Step 5. The feature vectors of peak value, energy, and sensor correlation coefficients of the testing samples are extracted.

Step 6. The extracted feature vectors from the testing samples are input into the trained MFSS-SVM classifier for classification, and the fall detection accuracy is calculated.

## 3. Experimental Verification

### 3.1. Experiment Setup

**Figure 2:**
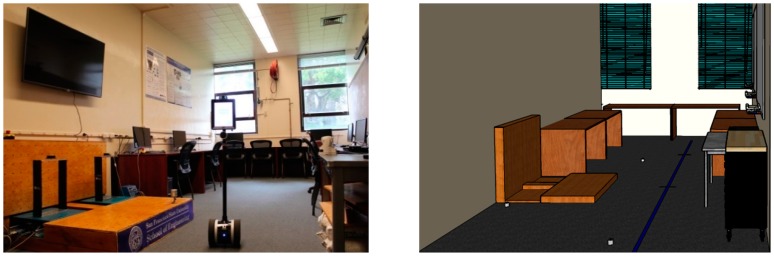
Intelligent structural hazard mitigation laboratory (iSHM) Lab at the San Francisco State University (Left: Picture; Right: 3D Model).

**Figure 3:**
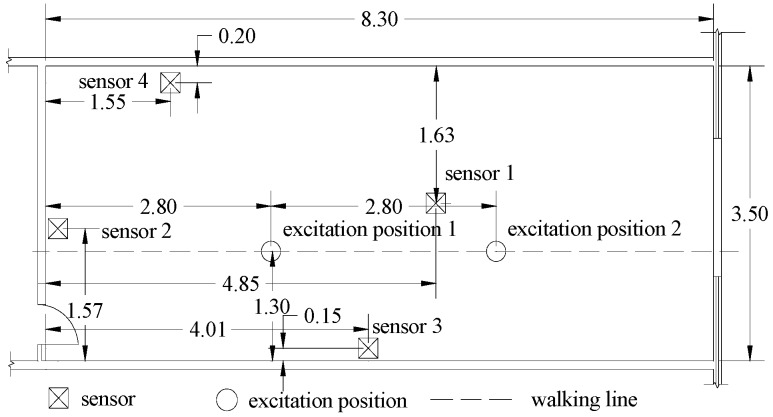
Sensor and excitation position arrangement in experiment (unit: m).

To test the proposed MFSS-SVM algorithm, experiments were performed in the intelligent structural hazard mitigation laboratory (iSHM Lab) at San Francisco State University (Figure 2) to collect vibration signals of human falling and other activities in daily life. Four PCB (PCB Piezotronics, Depew, NY, USA) high sensitivity uniaxial accelerometers (Model: 393B31; Sensitivity: 10.0 V/g; Frequency Range: 0.1 to 200 Hz) were used to measure the floor vibration signals. Considering the laboratory layout and the structural configuration of the slabs (e.g., underneath beams), three accelerometers were placed near the wall, and one was set near the center of the floor as shown in Figure 3. A National Instrument cDAQ-9171 (32-bit resolution) with a NI 9234 input module (24-bit resolution) was used as the data acquisition system. One thousand, six hundred and fifty-two hertz was chosen as the sampling frequency as it is the lowest sampling rate of the data acquisition system, and it is well above the frequency range of the human activities of interest.

**Figure 4:**
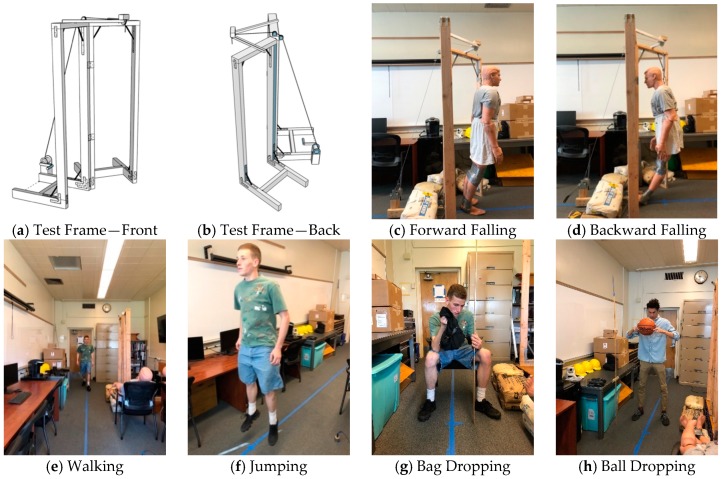
Experiment Setup.

Six types of activities, namely falling, walking, free jumping, rhythmic jumping, bag dropping, and ball dropping, were included in this study. The location of the excitation and the walking path are shown in Figure 3. The falling experiments were carried out with two Rescue Randy dummy human models (70 kg and 48 kg, respectively). These highly-imitated dummies include articulated joints and have weight distribution based on the human weight distribution chart. A wooden frame with a pulley system was constructed to release the dummies, ensuring the replicability of each experiment (Figure 4a,b). The falls were divided into forward falls and backward falls based on the direction of the falls (Figure 4c,d). Sixty-five repeated experiments were carried out at each excitation point for each falling direction, with a total of 520 falls conducted (2 dummies × 2 excitation points × 2 directions × 65 falls each). Two volunteers with a weight of 69 kg and 78 kg, respectively, performed 65 free walking experiments following the designed route (blue line in Figure 2 and dash line in Figure 3), with a total of 130 walking experiments (Figure 4e). The same volunteers also carried out 65 free and rhythmic jumping experiments (Figure 4f), respectively, at each excitation position, totaling 260 experiments for free and rhythmic jumping each. The rhythmic jumping was guided by a metronome, which was set at 90 beats per minute (1.5 Hz). Two bags with a mass of 5 kg and 10 kg at 0.6 m height were used in the bag dropping tests (Figure 4g). Each bag was subjected to 65 repeated experiments at each excitation position, totaling 260 times. Basketball, with a mass of 0.6 kg, was used for the ball dropping tests (Figure 4h). The height of the dropping was 1.45 m and 2.10 m, respectively. A total of 260 basketball fall tests (65 repeated experiments at each dropping position and height) were carried out. A summary of the experiments is provided in Table 1.

### 3.2. Results and Discussion

**Figure 5:**
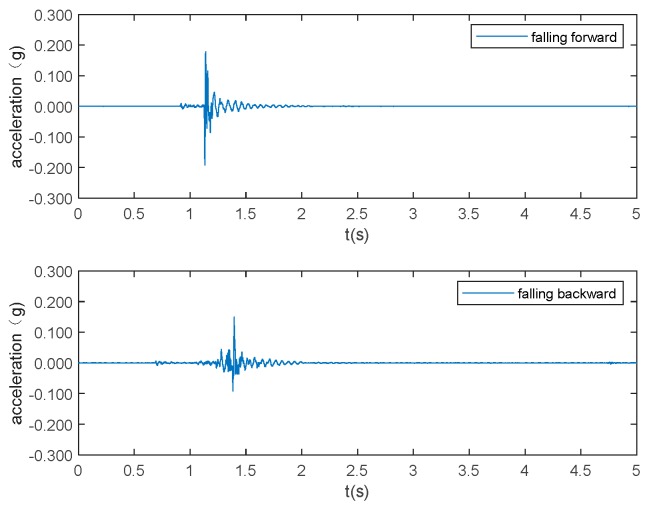
Dummy weighing 75 kg falls.

**Figure 6:**
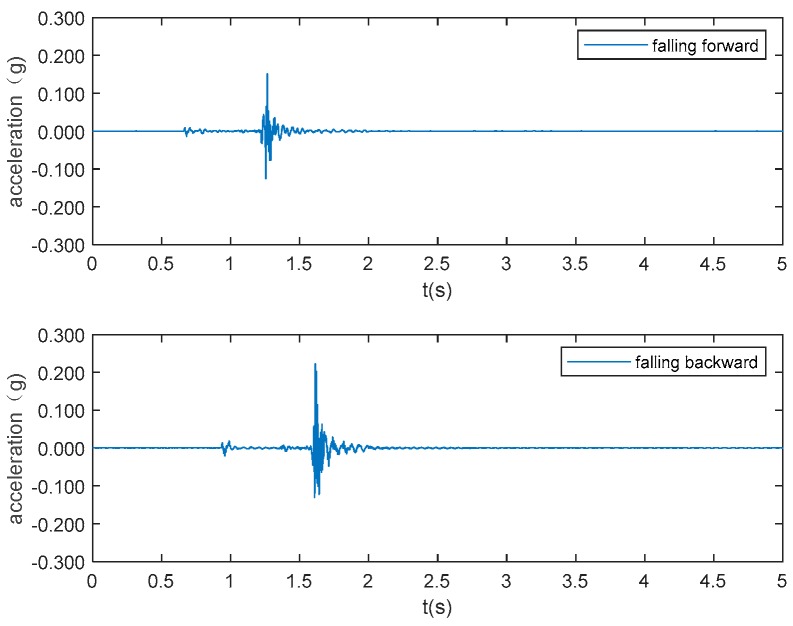
Dummy weighing 48 kg falls.

**Figure 7:**
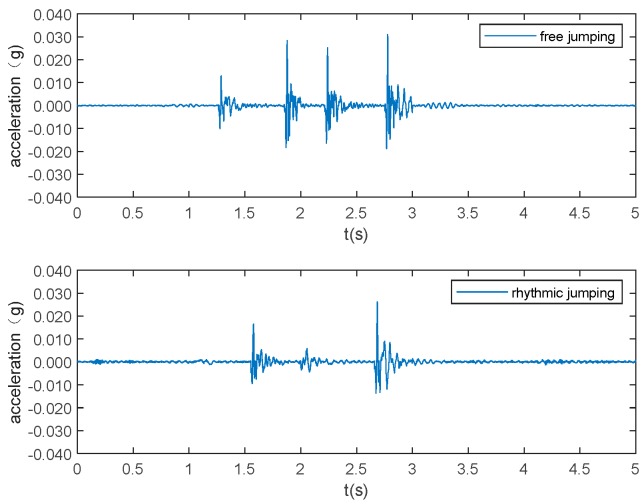
Volunteer weights 69 kg jumping.

**Figure 8:**
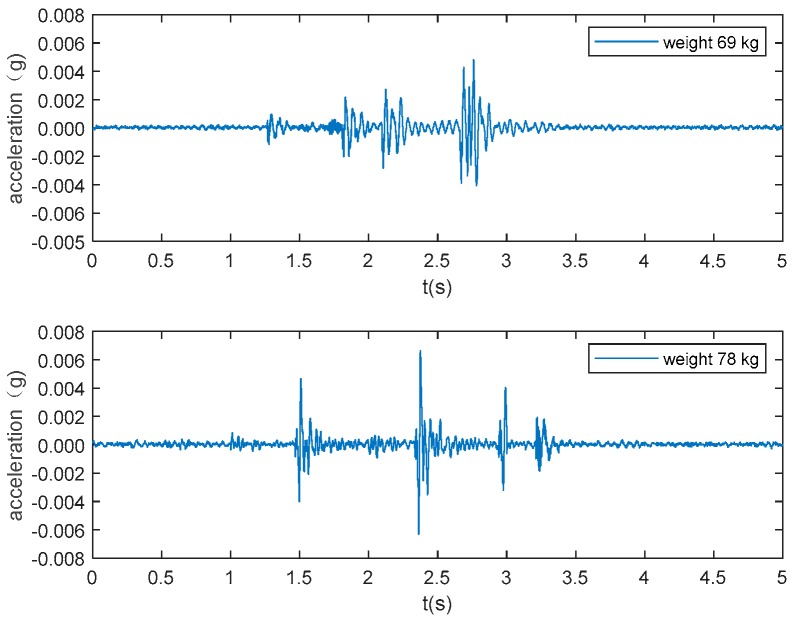
Volunteers (78 kg and 69 kg) walking.

**Figure 9:**
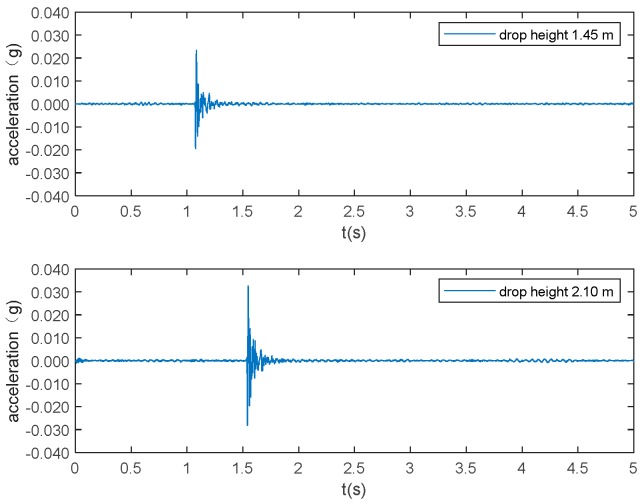
Ball (0.6 kg) drops.

**Figure 10:**
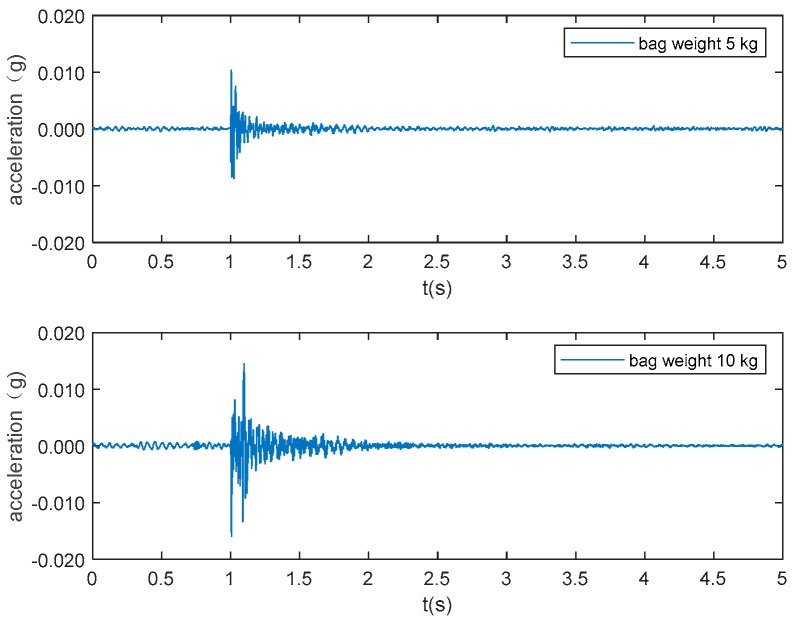
Bag (5 kg and 10 kg) drops.

The typical acceleration signals collected in the experiment from sensor 1 are shown in Figure 5 to 10 as examples. Figure 5 and Figure 6 show the measurements from the falling of the dummy models (75 kg and 48 kg) at the excitation position 1. Figure 7 demonstrates the acceleration signal when the volunteer jumped at the excitation position 1. Figure 8 is the acceleration signal when the volunteer (69 kg) walked along the walking line. Figure 9 and Figure 10 display the acceleration signals when the ball and bags with a weight of 5 and 10 kg fell at the excitation position 1.

The feature sets of peak value, energy, and sensor correlation coefficient were obtained from the experimental database. Each feature set contains 520 events of falling data and 1,170 events of other activities data. Typical feature vectors of various events at excitation position 1 are shown in Table 2, Table 3 and Table 4. Note that these results are merely one example among the large datasets, and they do not represent the statistical results.

Previous research has proved that more accurate and diverse base classifiers can achieve a better ensemble result [43]. As previously described in Section 2.3, the proposed MFSS-SVM algorithm composed of the three base SVM classifiers and the framework shown in Figure 1 was used to integrate them for fall classification. The application process has two main phases: First, three different base classifiers corresponding to three selected signal features (i.e., peak value, energy, and sensor correlation coefficient) were generated in a parallel manner; then the majority-vote model was utilized as a combination strategy to integrate them for forming the multiple base classifiers.

The testing datasets were randomly divided into training and testing datasets with a 7/3 ratio. In the testing data, three feature sets were extracted using the measurements from all the four sensors shown in Figure 3. Therefore, the input vectors to the classifiers are four dimensions for both the peak feature set and energy feature set, and six dimensions for the correlation coefficient feature set, respectively. Within the training datasets, data were further randomly selected for labeled and unlabeled categories. Labeling rates of 20%, 40%, 60%, and 80% were employed to investigate the performance of the MFSS-SVM. Data were processed using the SVM Toolbox in MATLAB R2016b. The fall detection results are shown in Table 5. The initial accuracy (detection results integrated from three feature vectors using SVM; outputs from step 2 of the proposed framework), final accuracy (detection results using MFSS-SVM; outputs from step 6 of the proposed framework), and time consumption (the time needed for training the model) were selected as indexes to evaluate the algorithm performance.

From Table 5, it can be observed that the accuracy of fall recognition using the MFSS-SVM was significantly improved with the increase of labeling rates (20–80%), from 90.53% to 98.74%, respectively. The performance increase comes with a cost. The time consumption of the program increased from 195 s to 299 s for labeling rate of 20% to 80%. It is worth noting that once the model is trained, the execution time needed to identify the falling event from the new data is less than 1s and only a small training set is required, which demonstrates the potential of a real-time classification algorithm for quick fall detection application.

**Figure 11:**
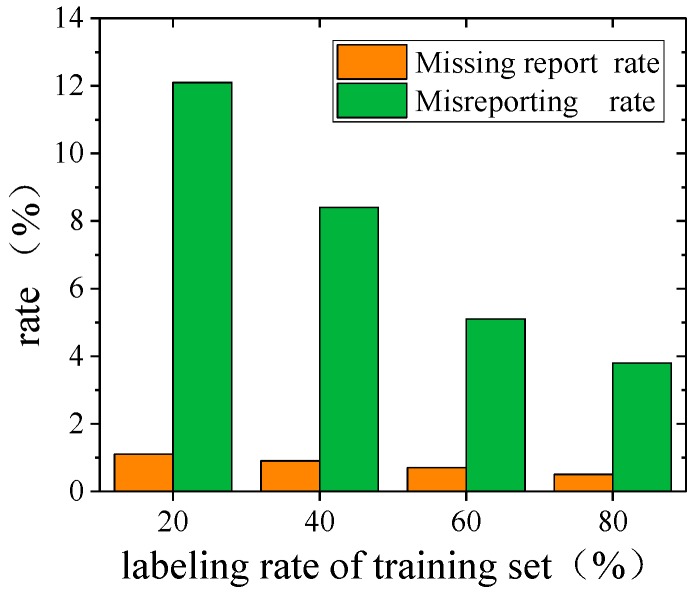
Missing report rate and misreporting rate of falling events.

**Figure 12:**
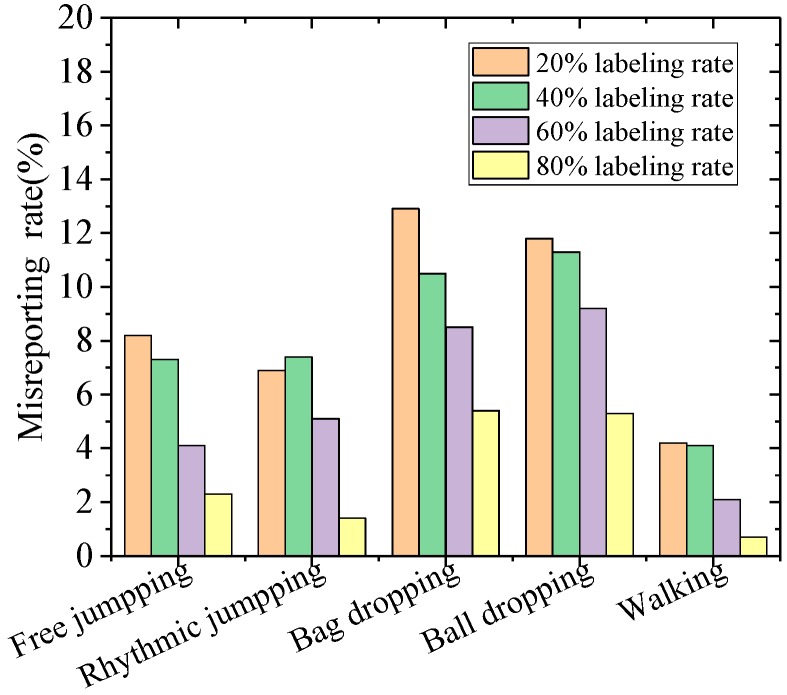
Misreporting rate of SVM-D-R method for non-fall events.

Figure 11 plots the missing report rate [44] of the proposed MFSS-SVM framework for detecting falls among various events in the experimental database. The miss detection rate was calculated as the ratio of falling events being mistakenly reported as non-falling to the total number of falling events in the testing datasets. It can be seen that the missing report rate in different labeling rates was marginal, with an average of 0.8%, while the false alarm (namely misreporting) rate [45] was also low, with an average of 7.4%. The false alarm rate was calculated as the ratio of the non-falling event being mistakenly reported as falling to the total number of non-falling events in the testing datasets. Figure 12 further shows the false alarm rate of falling in terms of different activities. It can be observed that the most confusing activities to the algorithm in identifying falls were bag dropping and ball dropping as they have the similar characteristics of an impulse impact as falling.

## 4. Comparison Study with the Benchmark Problem

To further investigate the performance of the proposed MFSS-SVM algorithm, a benchmark study by Madarshahian et al., [13] was adopted to serve as a baseline. Since falls were not part of the experiments in the benchmark database, it needs to be first expanded. In this section, a human fall load model was first proposed and validated using the measured data from experiments, and then the model was applied to the benchmark experimental settings to generate falling data and add it to the benchmark database.

### 4.1. Benchmark Problem

**Figure 13:**
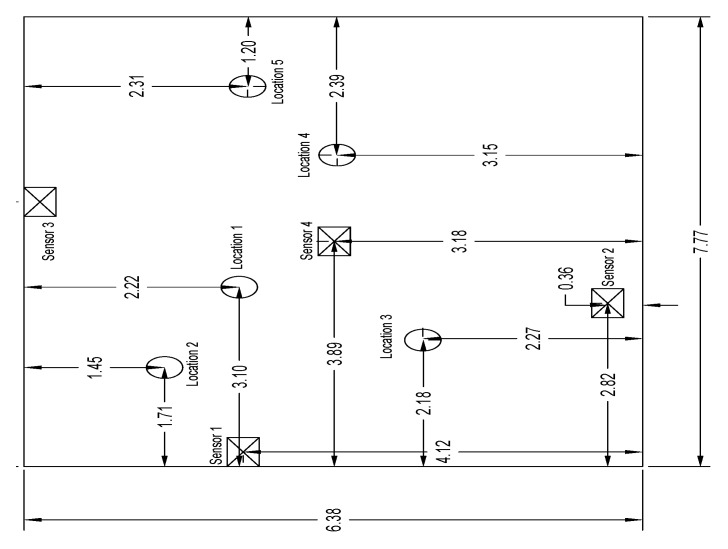
Sensor and impact locations in benchmark experiment (unit: m) [12].

In the benchmark problem, human activity recognition was performed using acquired floor vibration signals. An algorithm based on an autocorrelation function was used to identify the events. In that study, floor vibrations caused by different activities, including plastic bag dropping, basketball ball dropping, and human jumping (shown in Table 6), were collected at a laboratory setting. The laboratory is located on the second floor of a two-story steel structure. Figure 13 shows the five excitation and four sensor locations.

### 4.2. Human Fall Load Simulation

#### 4.2.1. Fall Load Model

To expand the benchmark problem and add the falling datasets, a human falling model needs to be first defined. According to the state of consciousness during the process, human falling can be divided into conscious and unconscious falls. Without completely losing consciousness, humans will subconsciously use the knee, arm, and other parts of the body to land on the ground to reduce injury. However, during unconscious falls, the person instantly collides with the ground without any protection, which would have a higher potential for severer injury. This study mainly focused on unconscious backward fall caused by ground slipperiness, tripping, loss strength of leg muscles, and so on. In addition to its potential greater impact and thus larger significance and value of research, it was selected because the unconscious falling can be simulated by anthropomorphic dummies with easier controllable experiment configurations. In the experimental study of a conscious fall involving real humans, sponge pads laying on the floor were often used to reduce the potential injury to the subjects [46]. This not only increases the complexity by introducing additional dynamics but also weakens the vibration signal of the floor to a certain extent.

**Figure 14:**
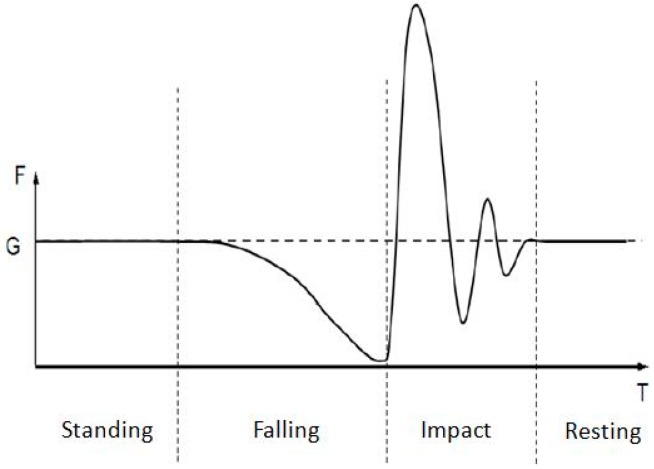
Floor force during the process of falling [46].

During the unconscious falling process, the external forces acting on the human body are gravity and the supporting force from the ground. The representative supporting force of an unconscious falling is shown in Figure 14. Based on the nature of human falls, the process can be generally divided into four stages: standing, falling, impact, and resting [47].

**Figure 15:**
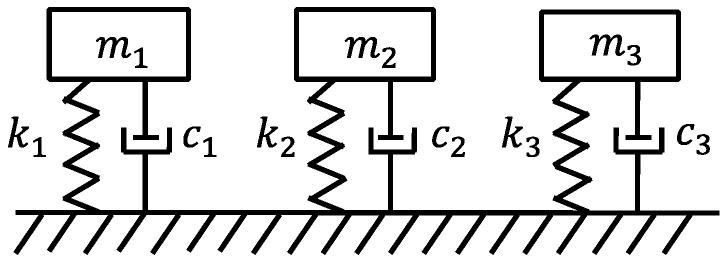
Model of a human body under vertical vibration [46].

A two-second unconscious falling can be divided as follows:

(1) Standing Stage (0–0.25 s): the gravity and supporting force of the ground are in a state of equilibrium (F = G).
(2) Falling Stage (0.25–0.75 s): the floor force has a parabolic distribution. At the end of this stage, the body is up in the air (no contact to the ground), and the force acting on the floor is zero.
(3) Impact Stage (0.75–1.75 s): the human body is in a supine position. The vibration state and force equilibrium of the human body after falling can be analyzed through physics. The international standard ISO 5982:1981 (1985) provides an impact point acceleration impedance model of the whole body under the vertical vibration. The model is shown in Figure 15. m_1_, m_2_, and m_3_ are the concentrated mass of the hip, back, and head, respectively. k_1_, k_2_, k_3_, c_1_, c_2_, and c_3_ are the corresponding stiffness and damping coefficients, respectively.
(4) Resting Stage (1.75–2 s): the human body is in a state of resting. In this state, the body has full contact with the ground, and its force to the ground is equal to the body’s weight.

To solve the force induced by the human body, the body vibration in the impact stage is simplified as a free vibration of three single-degree-of-freedom with damping under given initial conditions. The system equation of motion is established as

(6) $$m_{i}{\ddot{x}}_{i}+c_{i}{\dot{x}}_{i}+k_{i}x_{i}=0.$$

The initial conditions for the equation of motion are defined as

(7) $${\ddot{x}}_{i}\left(0\right)=g\;and\;{\dot{x}}_{i}\left(0\right)=\sqrt{2gh},$$

where h is the distance from the body center of gravity to the ground when the person is standing, where i = 1, 2, 3 for each degree-of-freedom. By substituting the equivalent mass, stiffness, and damping parameters of the hip, back, and head into the model, the equation of motion can be used to solve for the acceleration. Then, according to equilibrium, the floor force F can be obtained as

(8) $$F=\overset{3}{\underset{i=1}{\sum }}m_{i}{\ddot{x}}_{i}-m_{i}g.$$

**Figure 16:**
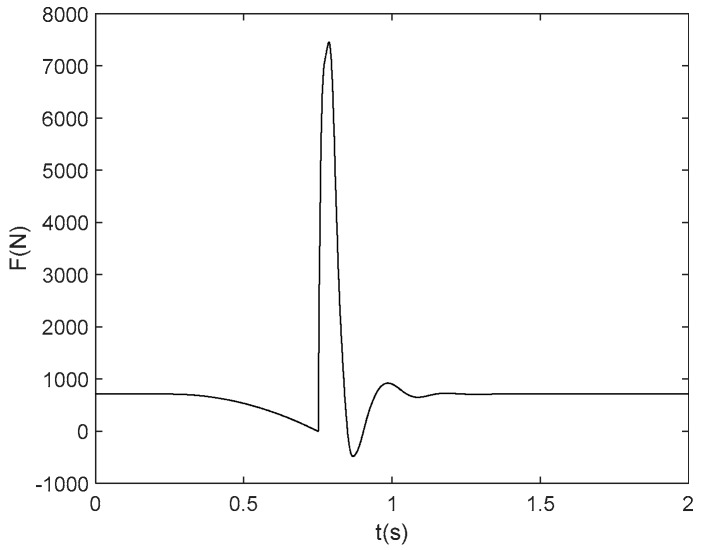
Floor force during the process of human falling (weight 71 kg).

In 1981, the international standard organization established an impact point impedance, in which the data was collected from 12 volunteers weighted between 62.2 kg to 104 kg. The final model parameters were determined by averaging the acceleration impedance curves; a more detailed explanation can be found in document [46]. A typical floor force during the process of human falling (weight 71 kg) is shown in Figure 16.

#### 4.2.2. Falling Load Model Verification

**Figure 17:**
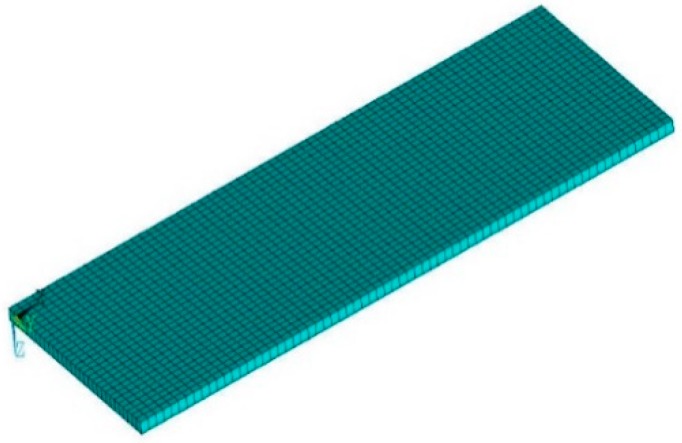
Finite element (FE) model of experimental laboratory floor.

Numerical analyses were performed using finite element (FE) models of the laboratory to verify the performance of the fall load model. An FE model of the floor in the iSHM Lab, as shown in Figure 17, on which the experiments were performed, was developed using ANSYS. The reinforced concrete (RC) slab was modeled using 2D shell elements. The RC beams in the floor were explicitly modeled with 1D elements, while the walls and columns were represented by constraints. Linear link elements were utilized to connect the RC beams to the concrete slab.

**Figure 18:**
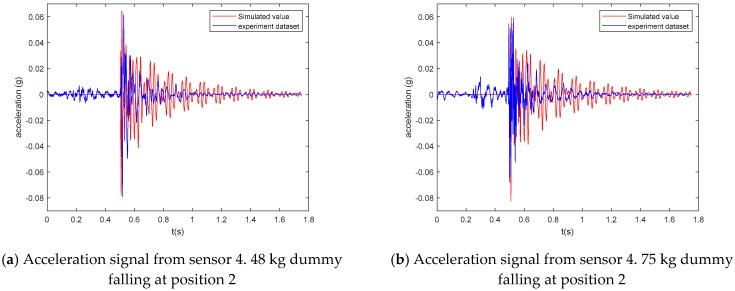
Comparison of simulated acceleration and experimental acceleration time history.

The dynamic response of the floor under point loads that were described in Equations (6)–(8) was evaluated using the developed FE model. The point loads (48 kg and 75 kg) were acting at the aforementioned dummy experiment positions. The vertical accelerations of the floors were obtained through a modal superimposition linear dynamic analysis. The comparison results between the FE simulation and the experimental testing of the typical floor vibration time-history are shown in Figure 18. A more detailed comparison is provided in Table 7.

As seen in Table 7, the FE simulations provided very similar vibrational performances and peak acceleration values as the experimental measurements. The falling process can be clearly divided into four stages as proposed, which confirmed that the simulated human falling load can be used to generate the falling datasets for expanding the benchmark database.

#### 4.2.3. Benchmark Database Expansion

**Figure 19:**
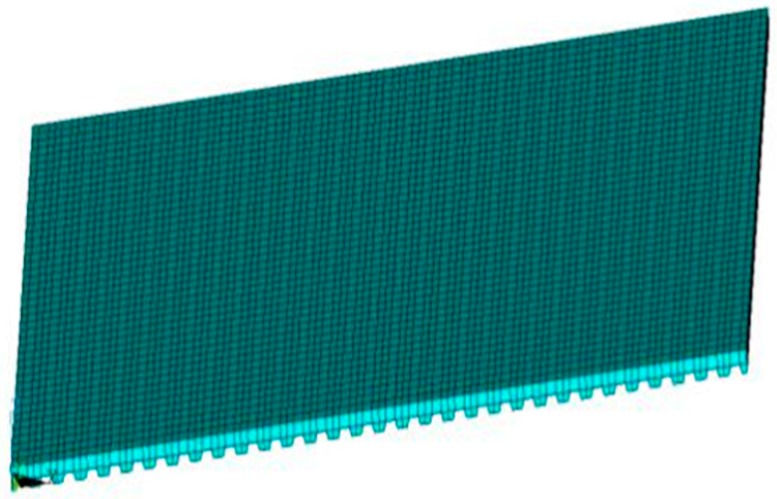
FE model of benchmark laboratory floor.

After the validation of the proposed human fall load model, it was used to create the dynamic excitations for inputting into the FE model of the concrete slab floor (see Figure 13) in the benchmark problem, as shown in Figure 19. In the slab FE model, the floor beams were explicitly modeled, whereas the walls and columns were considered as constraints. The steel beams of the laboratory floor were modeled with 1D elements, whereas 2D shell elements were used to model the concrete slab. Linear link elements were adopted to connect the steel beams to the concrete slab. The fundamental frequency of the FE floor model was 16.659 Hz, which closely matches (4.11% difference) with the 16.0 Hz measured from the experimental data reported by Madarshahian et al. [13]. The floor vibration responses of the FE floor model at the sensor locations in the benchmark problem under the simulated human falling loads were utilized to expand the benchmark database.

The modified database contains two types of events, the newly added falling events, and daily activities, including bag dropping, ball dropping, free jumping from the original benchmark problem database. The data structure of the new fall recognition database based on the benchmark problem is shown in Table 8.

### 4.3. Fall Detection Results

There is a total of 9200 datasets with 1725 falling and 7475 daily activities events in the expanded database. These data were divided into training and testing datasets with a ratio of 5.6/4.4 to evaluate the performance of the proposed MFSS-SVM algorithm over the one used in the benchmark program. Within the training datasets, data were randomly selected for labeled and unlabeled categories. Labeling rates of 20%, 40%, 60%, and 80% were investigated to evaluate the performance of proposed MFSS-SVM algorithm. To ensure the generality, the analyses were repeated three times, and the results were averaged to obtain the final results.

In the benchmark problem, an algorithm based on the difference of the acceleration autocorrelations was used to classify the various events. In this study, the two statistical measures, namely sensitivity and specificity, were adopted to examine the performance of the algorithms. The sensitivity criterion intends to measure the percentage of actual falling that are correctly identified as falling events while the specificity evaluates the percentage of non-falling events that are correctly identified as daily activities. The mathematical expressions of these two metrics are shown in Equations (9) and (10).

(9) $$\mathrm{Sensitivity}\;\left(S_{e}\right)=\frac{\sum True\;positive}{\sum True\;positive+\sum False\;negative},$$

(10) $$\mathrm{Specificity}\left(S_{p}\right)=\frac{\sum True\;negative}{\sum False\;positive+\sum True\;negative},$$

where true positive = correctly identified; false positive = incorrectly identified; true negative = correctly rejected; and false negative = incorrectly rejected.

The detection comparison results using the autocorrelation function implemented in the benchmark problem and the proposed MFSS-SVM algorithm with the expanded benchmark database are shown in Table 9. Results show that the MFSS-SVM algorithm works better than the autocorrelation function for all the different labeling rates in classifying the falling events, especially in terms of sensitivity. Compared to the algorithm in the benchmark problem, the sensitivity produced by the MFSS-SVM increased by 52.20%, 46.86%, 47.16%, and 43.74% for labeling rates of 20%, 40%, 60%, and 80%, respectively. In terms of specificity, the MFSS-SVM algorithm increased the performance by 0.20%, 1.19%, 0.62%, and 2.40%, respectively. The autocorrelation function was less successful in identifying the events, even using a high percentage of labeled samples.

## 5. Conclusions

Moving toward an accurate and real-time system for fall detection to improve the health outcomes of the older population, this paper investigated the use of an MFSS-SVM algorithm that takes advantage of the peak value, energy, and correlation coefficient of the accelerometer signal from floor vibrations as classification features to identify falling events.

The performance of the proposed algorithm was first validated with laboratory experiments among activities including simulated human falling using highly-imitated dummies, walking, free jumping, rhythmic jumping, bag dropping, and ball dropping. The results showed that the accuracy of the fall identification reached 90.53% with only 20% labeled data using the proposed algorithm. The accuracy was further improved from 90.53% to 98.74% with the increase of labeling rates (20–80%). The missing report (miss detection) and misreporting (false alarm) rates of using the proposed algorithm to detect falls were reported to be, on average, 0.8% and 7.4%, respectively. Bag dropping and ball dropping were identified as the most confusing activities to the algorithm in identifying falls as they have the similar characteristics of an impulse impact as falling.

To further illustrate the performance of the algorithm, a benchmark database was adopted and expanded to test the proposed algorithm’s ability to accurately identify falling. To generate fall data to add to the benchmark database, a human falling model was developed and utilized to create the dynamic inputs to an FE model of the concrete slab floor in the benchmark problem. By comparing the proposed algorithm with the algorithm used in the benchmark study, it showed that the proposed algorithm is capable of identifying falling events with significant improved accuracy and confidence, even with limited datasets available. Compared to the benchmark problem algorithm, the sensitivity of the MFSS-SVM increased by 52.20%, 46.86%, 47.16%, and 43.74% for labeling rates of 20%, 40%, 60%, and 80%, respectively. In terms of specificity, the MFSS-SVM algorithm increased the performance by 0.20%, 1.19%, 0.62%, and 2.40%, respectively.

In summary, results confirmed the performance of the proposed MFSS-SVM algorithm and demonstrated its great potential in identifying falls even with small training datasets and test nodes. Future works include further integration of the hardware to optimize sensor locations and the evaluation of the benefits of including other features in addition to peak value, energy, and sensor correlation coefficient, to characterize the strength and spread changes in the classifier. In addition, other classification methods, such as deep learning, are also being considered to further improve the performance for fall detection. The authors recognize the value of the datasets collected through this study to the community and are in the process of cleaning them up to share them in the near future.

## Figures and Tables

**Table 1:** Experimental Database.

Event Type	Event Description	Total Samples
Dummy falling	Dummies (weight 75 kg/48 kg) falling backward/forward	520
Walking	Volunteers (weight 69 kg/78 kg) walking freely	130
Free jumping	Volunteers (weight 69 kg/78 kg) jumping freely	260
Rhythmic jumping	Volunteers (weight 69 kg/78 kg) jumping at 1.5 Hz	260
Bag dropping	Bag (weight 5 kg/10 kg) dropping from 6 m	260
Ball dropping	Ball (weight 0.6 kg) dropping from 1.45 m/2.10 m	260

**Table 2:** Typical eigenvector at excitation position 1 of peak feature set.

Event	$MAX_{1}$	$MAX_{2}$	$MAX_{3}$	$MAX_{4}$
Bag falling	0.0666	0.0649	0.2480	0.0427
Ball falling	0.0254	0.0080	0.0398	0.0113
Free jumping	0.0506	0.0117	0.1008	0.0212
Rhythmic jump	0.0234	0.0042	0.0367	0.0069
Walking	0.0035	0.0011	0.0120	0.0017
Falling	0.1209	0.0474	0.2644	0.0525

**Table 3:** Typical eigenvector at excitation position 1 of energy feature set.

Event	$ENER_{1}$	$ENER_{2}$	$ENER_{3}$	$ENER_{4}$
Bag falling	3.2190	2.1728	9.8414	3.1104
Ball falling	2.4784	0.5775	3.0879	2.1906
Free jumping	4.8448	0.7042	7.6311	2.6972
Rhythmic jump	3.8713	0.6617	6.5570	2.5633
Walking	2.6530	0.4911	4.1279	2.0353
Falling	12.2471	2.8369	24.8655	8.2265

**Table 4:** Typical eigenvector at excitation position 1 of sensor correlation coefficient feature set.

Event	$CORR_{1}$	$CORR_{2}$	$CORR_{3}$	$CORR_{4}$	$CORR_{5}$	$CORR_{6}$
Bag falling	0.2877	0.4006	0.2220	−0.0653	−0.0696	0.1756
Ball falling	−0.1635	0.4765	0.0995	−0.0663	−0.2399	−0.1234
Free jumping	−0.0704	0.4933	0.2216	−0.0046	0.0348	−0.0846
Rhythmic jump	−0.0907	0.5835	0.2845	0.0327	0.0072	0.2690
Walking	0.2457	0.5865	0.3046	0.1826	0.2173	0.1778
Falling	−0.0050	0.6424	0.4750	0.0590	0.0787	0.3026

**Table 5:** Accuracy assessment of fall detection using a multi-features semi-supervised support vector machine (MFSS-SVM).

Labeled Training Dataset (%)	Initial Accuracy (%)	Final Accuracy (%)	Performance Increased (%)	Time Consumption (s)
20	83.53	90.53	8.38	195
40	84.91	92.61	9.07	221
60	88.40	95.54	8.07	258
80	91.42	98.74	9.18	299

**Table 6:** Data structure of benchmark database.

Event Type	Event Amplitude
Bag dropping	Drop height 1.45 m/2.10 m
Ball dropping	Drop height 1.45 m/2.10 m
Free jumping	Weight 80 kg/55 kg/85 kg

**Table 7:** Comparison of peak vertical acceleration from sensor 4 at position 2 (unit: g).

Experiment Dataset						Simulation
Dummy	Mean value	Variance	Median	Minimum	Maximum	
48 kg	0.0334	1.2826 × 10^−4^	0.0329	0.0152	0.0616	0.0643
75 kg	0.0382	8.5286 × 10^−5^	0.0376	0.0196	0.0622	0.0597

**Table 8:** Data structure of fall recognition database used for comparison.

Event Type	Event Description	Data Sources
Falling	Weight 60 kg/70 kg/80 kg	Simulation in the paper
Bag falling	Drop height 1.45 m/2.10 m	Benchmark database
Ball falling	Drop height1.45 m/2.10 m	Benchmark database
Free jumping	Weight 80 kg/55 kg/85 kg	Benchmark database

**Table 9:** Comparison of fall detection between the MFSS-SVM and benchmark algorithm (%).

Algorithm	Raito of Labeled Samples to Training Samples							
	20%		40%		60%		80%	
	$S_{e}$	$S_{p}$	$S_{e}$	$S_{p}$	$S_{e}$	$S_{p}$	$S_{e}$	$S_{p}$
Autocorrelation	54.23	89.21	58.45	92.03	61.35	93.21	63.47	93.24
MFSS-SVM	82.54	89.41	85.84	93.13	90.28	93.79	91.23	95.48
