# Correction: Liu et al. Detection of Human Fall Using Floor Vibration and Multi-Features Semi-Supervised SVM. *Sensors* 2019, *19*, 3720

**DOI:** 10.3390/s21113841

**Published:** 2021-06-02

**Authors:** Chengyin Liu, Zhaoshuo Jiang, Xiangxiang Su, Samuel Benzoni, Alec Maxwell

**Affiliations:** 1 Department of Civil and Environmental Engineering, Harbin Institute of Technology, Shenzhen 518055, China; chengyin.liu@hit.edu.cn (C.L.); suxiangxiang@stu.hit.edu.cn (X.S.); 2 School of Engineering, San Francisco State University, San Francisco, CA 94132, USA; sbenzoni@mail.sfsu.edu (S.B.); awm@mail.sfsu.edu (A.M.)

The authors wish to add one reference [1] to this paper [2].

On page 1, the original sentences are as follows: Progress toward new methods without cameras has been made through the use of accelerometer sensors mounted on the structure to capture floor vibration for human activities detection [13–15].

Correction to be: Progress toward new methods without cameras has been made through the use of accelerometer sensors mounted on the structure to capture floor vibration for human activities detection [13–16].

The sequential references originally in the paper (i.e., [16–47]) will shift by one (i.e., [17–48]).

The authors apologize for any inconvenience caused and state that the scientific conclusions are unaffected. The original article has been updated.
